# Silicone Oil Adhesion to Hydrophobic Acrylic Intraocular Lenses: A Comparative Laboratory Study of a New versus an Established Hydrophobic Acrylic Intraocular Lens Material

**DOI:** 10.1155/2021/1387987

**Published:** 2021-11-10

**Authors:** Gerd U. Auffarth, Hui Fang, Qiang Wang, Fritz Hengerer, Ramin Khoramnia, Hyeck-Soo Son, Sonja Schickhardt

**Affiliations:** ^1^The David J Apple International Laboratory for Ocular Pathology, Department of Ophthalmology, University of Heidelberg, Heidelberg 69120, Germany; ^2^Department of Ophthalmology, Third Affiliated Hospital, Wenzhou Medical University, Rui'an, Zhejiang 325200, China; ^3^Eye Hospital at Bürgerhospital Frankfurt, Nibelungenallee 37-41, 60318, Frankfurt, Germany

## Abstract

**Background:**

In vitro assessment of silicone oil adhesion to a new hydrophobic acrylic intraocular lens (IOL) material, the Clareon model CNA0T0, compared with the established AcrySof model SN60WF was carried out.

**Methods:**

Silicone oil adhesion was assessed for two types of IOLs, Clareon CNA0T0 (*n* = 10) and AcrySof SN60WF (*n* = 10). Lenses were immersed in an aqueous sodium chloride solution for 12 hours and then incubated at room temperature in silicone oil for 12 hours. The lenses were washed with distilled water and photographed at 25x magnification using a microscope. The percent coverage was calculated by dividing the area of oil coverage by the total surface area of the lens.

**Results:**

Silicone oil adhesion to the surface of the CNA0T0 lens ranged from 4% to 22%, with a mean ± SD coverage of 8% ± 4%. Silicone oil adhesion to the surface of the SN60WF lens ranged from 1% to 17%, with a mean coverage of 9% ± 4%. The silicone oil adhesion of CNA0T0 was equivalent to that of SN60WF (*P* > 0.05).

**Conclusions:**

The new Clareon model CNA0T0 IOL has silicone oil adhesion and interaction that are equivalent to the established AcrySof IOL.

## 1. Introduction

Silicone oil is used as an intraocular tamponade in vitreoretinal surgery to reduce fluid flow through retinal tears, preventing recurrent detachment [1, 2]. Patients with IOLs who require vitreoretinal surgery may experience additional postoperative complications from silicone oil tamponades [3]. Silicone oil can adhere to the surface of the IOL, leading to visual disturbances and deterioration of visual quality [4–7]. Removal of silicone oil from certain lens types can be accomplished with mechanical methods; however, these require additional invasive procedures [3].

Oil adhesion is a relatively rare surgical complication, first reported in case studies of explanted silicone IOLs in the 1990s [3, 5, 7]. Subsequent in vitro studies demonstrated that the degree of silicone oil adhesion depended on the biomaterial properties of the IOL, primarily the hydrophobicity of the lens material [3, 8]. Acrylic polymer lenses have been shown to have less silicone oil adhesion than silicone-based models as adhesion is proportional to hydrophobicity and silicone is more hydrophobic than acrylic material [8].

As new IOL materials are developed, in vitro assessment of silicone oil adhesion can evaluate its clinical impact. Interaction of silicone oil with IOL material is a particularly important consideration for pseudophakic patients at risk of retinal tears or proliferative vitreoretinopathy [3, 7].

An innovative hydrophobic acrylic IOL, the Clareon CNA0T0 (Alcon Laboratories, Inc., Fort Worth, TX, USA), has recently gained CE mark approval. The Clareon CNA0T0 lens is made of a novel hydrophobic acrylic polymer with a water content of 1.5% at 35°C and a refractive index of 1.55. BCNA0T0 has a full 6.0 mm functional biconvex aspheric optic with an overall length of 13.0 mm [9]. The CNA0T0 lens is a single-piece design with STABLEFORCE haptics that is based on the AcrySof SN60WF IOL design and provides predictable mechanical stability [9–11]. This study evaluated the silicone oil adhesion properties of the Clareon CNA0T0 IOL compared with the adhesion properties of the AcrySof SN60WF IOL.

## 2. Materials and Methods

### 2.1. Silicone Oil Adhesion Procedure

**Figure 1:**
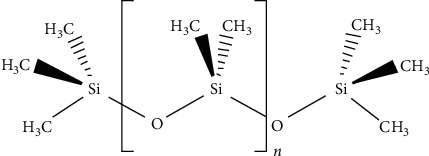
Structure of Siluron 5000.

Intraocular lenses of each model (*n* = 10 + 20 D CNA0T0; *n* = 10 + 20 D SN60WF) were immersed in microcentrifuge vials containing 0.9% aqueous sodium chloride (NaCl) solution (Braun, Melsungen, Germany) at room temperature for 12 hours to simulate aqueous in vivo conditions [3]. The lenses were removed from the sodium chloride solution and then immersed in 5000 centistoke silicone oil [3, 4], Siluron 5000 (Ultrapurified Silicone Oil, Geuder AG, Heidelberg, Germany, Figure 1), for 12 hours at room temperature.

**Figure 2:**
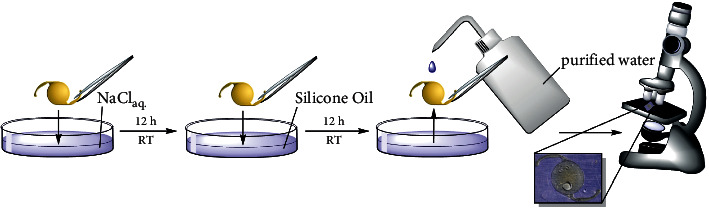
Investigation process of the silicone oil adhesion procedure.

After immersion in silicone oil, the lenses were rinsed and immersed in distilled water to aid visualization of the silicone oil coverage as shown in Figure 2 [3].

### 2.2. Coverage Calculations

**Figure 3:**
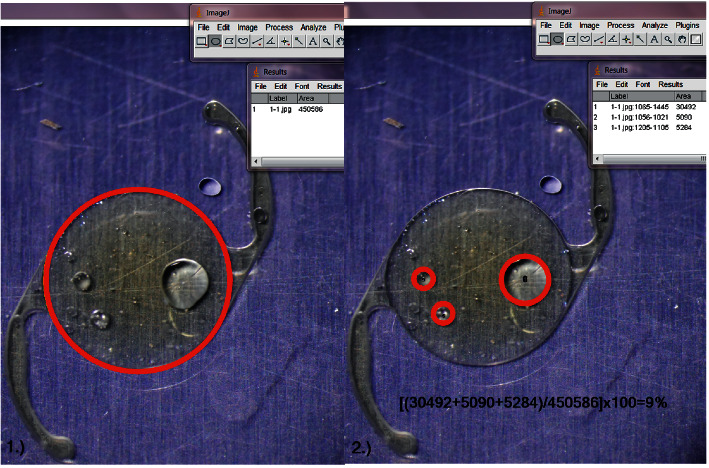
Evaluation with ImageJ for silicone oil adhesion. (a) The whole area of the central optic is given by 450586 pixel^2^. (b) The silicone oil adhesion on the IOL is given by (1) 30492 pixel^2^; (2) 5090 pixel^2^; (3) 5284 pixel^2^. The result is the difference between the area wetted with silicone oil and the total area of the optic.

Silicone oil coverage was evaluated by photographing each lens with an INFINITY 1-2CB camera (Lumenera Corporation, Ottawa, ON, Canada) at 25x magnification under an EMZ-8TR Trinocular Zoom Stereo Microscope (Meiji Techno, Saitama, Japan). Quantitative measurements of silicone oil coverage of the IOLs were made using image analysis software (ImageJ, US National Institutes of Health, Bethesda, Maryland, USA, https://imagej.nih.gov). The evaluation procedure is shown in Figure 3.

The percent coverage was calculated by dividing the area covered by oil by the area of the lens. This analysis was performed separately for the anterior and posterior sides of each lens.

### 2.3. Statistics

One-way ANOVA was conducted to compare the total anterior and posterior silicone adhesion of the CNA0T0 to SN60WF IOL model and to also compare the anterior or posterior of CNA0T0 to that of the corresponding surface of the SN60WF IOL (Minitab 17, State College, PA, USA).

## 3. Results

### 3.1. Silicone Oil Adhesion

The CNA0T0 lens silicone oil adhesion ranged from 4% to 22%, with a mean ± SD coverage of 8% ± 4%. Silicone oil adhesion to the surface of the SN60WF lens ranged from 1% to 17%, with a mean ± SD coverage of 9% ± 4%.

The results for each IOL are summarized in Table 1.

**Figure 4:**
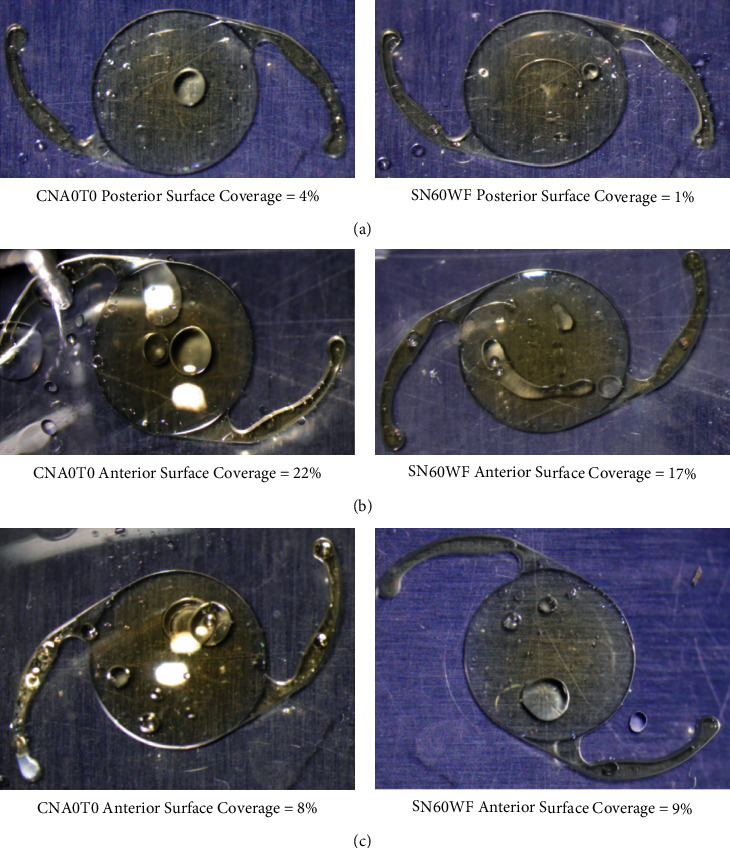
Digital images of silicone oil coverage on the IOL: (a) lowest coverage, (b) highest coverage, and (c) mean coverage (representative examples). IOL: intraocular lens.

Representative digital images depicting the lowest, highest, and mean percent oil coverage for each of the 2 IOLs tested are shown in Figure 4.

The silicone oil adhesion of CNA0T0 was equivalent to that of SN60WF (*P* > 0.05). Additionally, silicone oil adhesion on the anterior surfaces and posterior surfaces of CNA0T0 and SN60WF was equivalent (*P* > 0.05). Most of the silicone oil was removed after 2 minutes of irrigation/aspiration following the postimmersion observations.

## 4. Discussion

Vitreoretinal surgery is performed to address complex conditions such as retinal tears and detachment, proliferative vitreoretinopathy, and diabetic retinopathy; it is often facilitated by a tamponade agent injected to replace the vitreous fluid [7, 12, 13]. Tamponades help prevent further damage by reducing the flow of fluid through open tears, while the repaired or reattached retina heals [1, 2]. Gas or silicone oil can be used as retinal tamponades; the benefits and disadvantages of these materials have been discussed in a recent review of comparative studies [1]. The major benefit of the gas tamponade is that it spontaneously dissipates, while silicone oil removal requires an additional surgical intervention [1]. Although some studies have shown higher surgical success rates and significantly better visual outcomes with the use of silicone oil compared with a gas tamponade, the choice of tamponade agent ultimately depends on individual factors, such as the classification of retinal detachment [2].

In the 1990s, a rare clinical complication from the use of silicone oil was reported in several case studies [5, 7]. Pseudophakic subjects with implanted silicone IOLs required vitreoretinal surgery with a silicone oil tamponade and subsequently experienced decreased visual acuity and visual aberrations. Surgeons observed silicone oil droplets adhered to the lenses; attempts to remove oil with vitrectomy instruments and aspiration were unsuccessful [5]. Evaluation of the explanted lenses in aqueous solution showed a thick coating of silicone oil that was not removable by mechanical pressure with an injected viscoelastic device [7]. Scanning electron microscopy demonstrated the extent of oil adherence to silicone IOLs. One of the explanted IOLs showed approximately 80% oil coverage of the lens surface [7]. The complications of silicone oil adherence, including visual disturbances in patients and difficulty for the operating surgeon in visualization of the surgical field during vitreoretinal procedures, led to recommendations against implanting silicone IOLs in patients at high risk of vitreoretinal disease [3, 7].

Following the clinical case reports, in vitro studies were performed to assess silicone oil adherence to various IOL materials and to crystalline lenses from human cadaver eyes [4, 14]. Adhesion to human crystalline lenses was not previously reported to cause clinically significant visual problems, and the in vitro study of crystalline lenses showed a mean ± SD adhesion of 11% ± 6% [4]. Oil was easily removed from human lenses by injection of a viscoelastic device. Four IOL biomaterials that showed comparable adhesion to human lenses would therefore not be expected to have clinically significant effects on visual acuity [4].

Silicone IOLs stored in oil for long periods of time (≥1 year) exhibited a chemical interaction between the lens polymer and oil, resulting in a continuous layer of oil on the surface of the lens [14]. In a study evaluating silicone oil adhesion to 7 different IOL materials, adhesion ranged from a mean ± SD of 9% ± 7% for heparin-surface-modified IOLs to 100% ± 0% for silicone IOLs [4]. All other lens biomaterials had significantly less adhesion than silicone lenses (*P* < 0.001) [4]. Hydrophilic acrylic IOLs, such as those with heparin surface modification, generally showed less silicone oil adhesion than hydrophobic acrylic IOLs due to the larger contact angle between silicone polymers and hydrophobic materials [3]. However, heparin coatings on IOLs are no longer common. Hydrophobic acrylic lenses (AcrySof) had mean adhesion of 34% ± 14% [4]. In a later study comparing multiple types of acrylic lenses, mean silicone oil adhesion to AcrySof was reported as 17% ± 3% [8].

In the current study, 8% to 9% silicone oil adhesion was observed for both hydrophobic acrylic IOL materials, indicating that the new Clareon CNA0T0 IOL has oil interaction equivalent to that of the AcrySof SN60WF lens. It is interesting to note that the Clareon CNA0T0 and AcrySof SN60WF IOLs had silicone oil adhesion comparable to that reported previously for the human crystalline lens (11%), and therefore may not cause significant visual disruption in patients who require silicone oil tamponades during vitreoretinal surgery [4].

5000 mPas silicone oil (Siluron^®^ 5000 (Ultrapurified Silicone Oil, Geuder AG, Heidelberg, Germany) was used in this study. It is the common silicone oil in our clinic. Other departments may use different oils, and other studies [8] used 1000 mPas silicone oil. Demonstrated by Senn et al. [15], no obvious differences between the viscosities of 1000 mPas and 5000 mPas silicone oils in terms of oil-lens interaction could be observed.

Also of interest is the decrease in reported silicone oil adhesion to AcrySof SN60WF lenses compared with earlier studies. Since its introduction in the 1990s, there have been improvements to the AcrySof material and lens manufacturing process that have led to a decrease in glistening density [16, 17]. The improvements in manufacturing may have altered the affinity of the AcrySof biomaterial for silicone oil, causing the decreased adhesion observed in this study. This in vitro study demonstrated equivalent silicone oil adhesion to the Clareon CNA0T0 lens compared with the AcrySof SN60WF lens. Clinical studies of the new generation of hydrophobic acrylic lenses may be needed to confirm that silicone oil adhesion and silicone oil opacification may now be regarded as an unlikely complication for cataract patients receiving the new hydrophobic lens.

## 5. Conclusion

Silicone oil adhesion to the surface of the new Clareon CNA0T0 lens ranged from 4% to 22%, with a mean ± SD coverage of 8% ± 4% (Table 1). Silicone oil adhesion to the surface of the established AcrySof SN60WF lens ranged from 1% to 17%, with a mean coverage of 9% ± 4% (Table 1). The silicone oil adhesion of CNA0T0 was equivalent to that of SN60WF (*P* > 0.05). Additionally, silicone oil adhesion on the anterior surfaces and posterior surfaces of CNA0T0 and SN60WF was equivalent (*P* > 0.05).

## Figures and Tables

**Table 1:** Percentage of silicone oil coverage on the intraocular lens (IOL).

IOL no.	AcrySof		Clareon	
	Anterior^*∗*^ (%)	Posterior^*∗*^ (%)	Anterior^*∗*^ (%)	Posterior^*∗*^ (%)
1	10	11	8	7
2	17	11	11	9
3	6	6	22	6
4	5	8	8	5
5	6	6	7	4
6	14	15	4	10
7	6	1	4	7
8	8	17	6	12
9	9	7	11	9
10	10	9	12	4

Mean ± SD^†^	9 ± 4		8 ± 4	

IOL: intraocular lens. ^*∗*^Results for each surface of a lens; adhesion was measured by looking first at 1 side and turning the lens over to measure the adhesion on the opposing surface. There was no expectation of a difference in adhesion between the 2 sides. ^†^Based on all assessments (10 anterior and 10 posterior).

## Data Availability

The data used to support the findings of this study are available from the corresponding author upon request.

## References

[1] Schwartz S. G., Flynn H. W., Lee W. H., Wang X. (2014). Tamponade in surgery for retinal detachment associated with proliferative vitreoretinopathy. *Cochrane Database of Systematic Reviews*.

[2] Vaziri K., Schwartz S. G., Kishor K. S., Flynn H. W. (2016). Tamponade in the surgical management of retinal detachment. *Clinical Ophthalmology (Auckland, N.Z.)*.

[3] Arthur S. N., Peng Q., Apple D. J. (2001). Effect of heparin surface modification in reducing silicone oil adherence to various intraocular lenses. *Journal of Cataract & Refractive Surgery*.

[4] Apple D. J., Isaacs R. T., Kent D. G. (1997). Silicone oil adhesion to intraocular lenses: an experimental study comparing various biomaterials. *Journal of Cataract & Refractive Surgery*.

[5] Kusaka S., Kodama T., Ohashi Y. (1996). Condensation of silicone oil on the posterior surface of a silicone intraocular lens during vitrectomy. *American Journal of Ophthalmology*.

[6] Rosca C., Munteanu M., Tamasoi I. (2016). Calcification of hydrophilic acrylic intraocular lens in eyes with silicone oil tamponade - an interventional case series report. *Acta Ophthalmologica*.

[7] Apple D. J., Federman J. L., Krolicki T. J. (1996). Irreversible silicone oil adhesion to silicone intraocular lenses. A clinicopathologic analysis. *Ophthalmology*.

[8] McLoone E., Mahon G., Archer D., Best R. (2001). Silicone oil-intraocular lens interaction: which lens to use?. *British Journal of Ophthalmology*.

[9] Lane S., Collins S., Das K., Maass S., Thatthamla I., Jain R. Evaluation of the mechanical behavior of a new single-piece intraocular lens as compared to commercially available IOLs.

[10] Lane S. S., Burgi P., Milios G. S., Orchowski M. W., Vaughan M., Schwarte E. (2004). Comparison of the biomechanical behavior of foldable intraocular lenses. *Journal of Cataract & Refractive Surgery*.

[11] Auffarth G. U., Schickhardt S., Wang Q., Fang H., Hengerer F. H. Laboratory evaluation of the new Clareon hydrophobic acrylic IOL material: biomaterial properties and capsular bag behavior.

[12] Falkner C. I., Binder S., Kruger A. (2001). Outcome after silicone oil removal. *British Journal of Ophthalmology*.

[13] Foster W. J. (2008). Vitreous substitutes. *Expert Review of Ophthalmology*.

[14] Stolba U., Binder S., Velikay M., Wedrich A. (1996). Intraocular silicone lenses in silicone oil: an experimental study. *Graefes Archive for Clinical and Experimental Ophthalmology*.

[15] Senn P., Schmid M. K., Schipper I., Hendrickson P. (1997). Interaction between silicone oil and silicone intraocular lenses: an in vitro study. *Ophthalmic Surgery, Lasers and Imaging Retina*.

[16] Thomes B. E., Callaghan T. A. (2013). Evaluation of in vitro glistening formation in hydrophobic acrylic intraocular lenses. *Clinical Ophthalmology*.

[17] Wang Q., Yildirim T. M., Schickhardt S. K. (2021). Quantification of the in vitro predisposition to glistening formation in one manufacturer’s acrylic intraocular lenses made in different decades. *Ophthalmology and Therapy*.

